# Strategies to enhance sexual health education for prevention of teenage pregnancy in Vhembe District, Limpopo Province: different stakeholder’s perspectives, a co-operative Inquiry qualitative protocol paper

**DOI:** 10.1186/s12978-023-01669-x

**Published:** 2023-08-18

**Authors:** Nombulelo V. Sepeng, Fhumulani M. Mulaudzi, Pfarelo Mathivha, Maurine R. Musie, Raikane J. Seretlo

**Affiliations:** 1 Department of Nursing Science, Faculty of Health Sciences, University of Pretoria, Tshwane, South Africa; 2 Department of Public Health, Faculty of Health Science, Sefako Makgatho University, Tshwane, South Africa

**Keywords:** Adolescent, Strategies, Sexual health, Pregnancy, Prevention

## Abstract

**Background:**

South Africa is reporting higher rates of adolescent pregnancy as compared to other countries. There are different types of interventions that are in place to address teenage pregnancy. However, these interventions were developed using top-down strategy without the inclusion of different stakeholders and adolescents which makes it hard to implement those interventions particularly in countries like South Africa. Hence, this study aimed to develop strategies to enhance sexual health education for prevention of teenage pregnancy in Vhembe District, Limpopo Province of South Africa.

**Methods:**

The research design of this study will be Co-operative Inquiry.The study will take place in Vhembe District in Limpopo province of South Africa through collaborating with a Non-governmental Organisation (NGO). The study population will be the teenagers and all the different stakeholders caring for teenagers in their areas of specialization. Purposive sampling will be used to sample the targeted participants of the study. The data collection method will be done in phases and focus group discussions will be used to collect data. Content analysis will be used to analyse data.

**Discussion:**

This study will add to the body of knowledge regarding the strategies that maybe used to enhance sexual health education for prevention of teenage pregnancy.

**Supplementary Information:**

The online version contains supplementary material available at 10.1186/s12978-023-01669-x.

## Background

Teenage pregnancy is a global public health concern that affects young girls as young as 12 years old. According Håkansson, Oguttu [1] there were about 16 million adolescent girls aged between 15 and 19 accounts to 11% of all births worldwide. Approximately 130,000 babies were born in South African public health facilities to girls aged 10–19 in 2019. The number of deliveries for girls of the same age group increased to 136,386 in 2020 [2]. Rural areas have significantly higher rates [3]. Currently in there are about 775 teenagers that are pregnant in Vuwani, Vhembe District in Limpopo province of South Africa. There are different factors resulting to higher rates of adolescent pregnancy. Lack of knowledge and/or ineffective use of contraception, peer group influence, low socioeconomic status, family instability, early marriage, and cultural permissiveness have all been regarded as the contributing factors contributing to higher rates of adolescent pregnancy in developing countries [4].

The socioeconomic consequences resulting from adolescent pregnancy are concerning for both mothers and their children. One disadvantage for mothers is that they are less likely to complete a high school diploma or a university degree, limiting their future employment opportunities [5]. Furthermore, adolescent pregnancy has been linked to an increase in pregnancy complications such as anaemia, hypertension, eclampsia, and prolonged or premature labour and/or low birth weight babies [6]. Other complications associated with adolescent pregnancy include dysfunctional labour, pregnancy-related infections, postpartum haemorrhage, premature membrane rupture [6]. Furthermore, adolescent mothers are at greater psychological risk than their older counterparts because they experience higher levels of stress, despair, depression, feelings of helplessness, low self-esteem, a sense of personal failure, and suicide attempts [7].

Based on the negative consequences of adolescent pregnancies on the adolescent, various interventions have been implemented to prevent pregnancies [8]. Some recurring ones in the literature include: the provision and use of effective and accessible family planning methods, the use of condoms, the use of an emergency pill, and abstaining during the ovulation phase [9]. Other interventions for the adolescent population include school health programs and sexuality/family life education [9]. Despite the implementation of these interventions, higher rates of adolescent pregnancy persist in South Africa, particularly in rural areas. According to the most recent District Health Barometer, for example, approximately 8% of all mothers who give birth in Vhembe health facilities are under the age of 18 [10].

Preventing adolescent pregnancy has received a lot of attention around the world, including in South Africa. The South African government implemented various interventions, including the use of free and easily accessible contraceptives among adolescents, the distribution of condoms, the introduction of school health programs on sexual education and reproductive health among adolescents, youth friendly services clinics, and the adoption of SDGs goals [11–13]. However, teen pregnancy including learners remains prevalent in South Africa.

Most of sexual health programs are developed using top-down approach. In that aspects most of the stakeholders are not included to develop the intervention strategies for prevention of pregnancy. Stakeholders responsible for prevention of teen pregnancy includes parents, community leaders, health care professionals and adolescents themselves. In support of this, Nabugoomu, Seruwagi [14] stated that recommendations encompassing family, community, and government involvement can ultimately empower girls, their families, and community members, while also supporting collective action to reduce teen pregnancy. As a result, there is a need to explore and describe community and stakeholder’s perspective regarding the development of sexual health education strategies that may be used to enhance for prevention of teenage pregnancy in Vhembe District, Limpopo province of South Africa.

## Study aims and objectives

The aim of this study is to develop strategies to enhance sexual health education strategies that can be used for prevention of adolescent pregnancy in Vhembe district, in Limpopo province of South Africa through following co-operative inquiry qualitative research methodology to inform future effective implementation of interventions used for prevention on adolescent pregnancy. The specific objectives of the study are:

1. To explore and describe stakeholder’s concerns and sexual health education needs for prevention of adolescent pregnancy.
2. To explore and describe the perceptions of stakeholders regarding the development of strategies to enhance sexual health education strategies that can be used for prevention of adolescent pregnancy.
3. To develop strategies to enhance sexual health education strategies that can be used for prevention of adolescent pregnancy.
4. To validate the strategies developed to enhance sexual health education for prevention of adolescent pregnancy.

## Methods/design

### Study design

A Co-operative Inquiry qualitative study using focus group discussions with different stakeholders that is nurses, psychologist, social workers, teachers, community members, parents and adolescents and a purposive non-probability sampling method will be used in this study. The focus group discussions will be conducted in four cyclical phases of Co-operative Inquiry that is reflection phase, planning phase, action phase and observation phase.

### Study setting

The study will take place in Vhembe district Limpopo Province of South Africa through collaborating with a Non-governmental Organisation. The name of the NGO is the Isa Mathivha Foundation is a registered Non-profit company which works to support the development of communities in the rural areas of Limpopo in Vuwani, Collins Chabane Municipality and the surrounding areas. It was established with the aim of promoting the development of young people, establish family’s self-help initiatives and life skills education. The NGO aims to shape the future of the communities and teenagers by initiating life skills and poverty alleviation through psychoeducation and development programs for teenagers, young adults, and other families towards creating opportunities to achieve the desires of their hearts.

### Study participants

Purposive sampling technique will be used identify and recruit different stakeholders such as adolescents, nurses, community members, teachers, psychologists, and social workers to participate in the study. Detailed eligibility criteria are given below:

### Eligibility criteria

#### Inclusion criteria

- Female adolescent that are not pregnant and pregnant at a time of data collection.
- Adolescent mothers that already given birth post 6 weeks at a time of data collection.
- Male adolescents who do not have children and male adolescent fathers.
- Adolescents aged 16 years to 19 years.
- Key informants who are directly or indirectly involved in adolescent related matters such as teachers, social workers, psychologists, nurses, parents, and community members.

#### Exclusion criteria

- The study will not include adolescents who gave birth before 6 weeks to avoid exposing them to harm unintentionally as well as to respecting their culture during puerperium.

### Data collection methods and procedure

Data will be collected in four phases. Focus group discussion will be used to collect data. Different stakeholders will have their own focus group discussion and they will be grouped in one group of 8–10 members in each group for a focus group discussion in Co-operative Inquiry Group (CIG). The adolescents will also have their own focus group discussion in CIG, and they will be grouped in 8–10 into members. The adolescents will be separated from other stakeholders to allow freedom of expression during data collection. One of the researchers will act as a moderator and take field notes to document what has been by the participants and note down their facial expressions during data collection. The researchers will use a tape recorder with the permission of the participants and the researchers will explain that the tape recorder is mainly used for the purpose of transcribing data after data collection.

In the first phase a group of co-researchers comes together to investigate a specific area of human activity. During the first phase, they discuss their interests and concerns and decide on the focus of their investigation [15]. In phase one the researchers and co-researchers will firstly explore and describe concerns and sexual health education needs of stakeholders for prevention of teenage pregnancy in Venda. Following that, in this phase, the researchers will explore and describe the perspectives of stakeholders regarding the development of strategies to enhance sexual health education for prevention teenage pregnancy in Vhembe District. The researchers will analyse data for phase 1 and present it to the participants in phase 2. This will assist the researchers and participants to reflect on what they have discussed to check if the information shared with the researchers is correctly captured and represent the true meaning of what they have shared with the researchers. After that researcher will begin with phase 2 of the study (Additional files 1, 2).

In the second phase, group puts their agreed-upon actions into action in their daily lives and at work: they initiate the actions and observe and record the results of their own and each other’s behaviour [15]. They may initially simply observe what happens to them in order to gain a better understanding of their experience; later, they may begin experimenting with new forms of action [15]. In this phase, strategies to enhance sexual health education for prevention of teenage pregnancy in Venda will be developed. The researchers will analyse data for phase 2 and present it to the participants in phase 3. This will assist the researchers and participants to reflect on what they have discussed to check if the information shared with the researchers is correctly captured and represent the true meaning of what they have shared with the researchers. After that researcher will begin with phase 3 of the study.

In the third phase the co-researchers fully immerse themselves in their experience. They may become more aware of what is going on and begin to see their experience in new light [15]. They may delve deeper into the experience, elaborating and developing superficial understandings. Alternatively, they may be drawn away from the original ideas and proposals and into new fields, unexpected action, and creative insights. In some ways, this phase is the touchstone of the inquiry method, and it is what distinguishes it from conventional research, because here people are deeply involved in their own experience, and any practical skills or new understandings will emerge from this experience [15]. In this phase, the researchers will share the analysed results for phase 3 in the meeting that will be organised with the participants. The participants will be given a chance to researcher to validate the developed strategies to enhance sexual health education for prevention of teenage pregnancy in Venda and the revisions will be made, if possible, on the developed strategies. Each focus group will last for an hour or half an hour.

### Data analysis

The audio recordings of the focus group discussions will be transcribed and then translated into English for standard content analysis [16–18]. The transcripts will not contain any identifying information. The transcripts will be uploaded into the NVivo 12 plus software for easy and organized data retrieval and analysis. Transcripts will be read several times to develop an interpretation of the participants’ concerns and sexual health education needs for adolescent pregnancy prevention, as well as their perceptions of the development of strategies to improve sexual health education strategies that can be used for adolescent pregnancy prevention. Data will be coded, compared, contrasted, and refined in an interactive process to produce emergent themes. In order to address data gaps and seek information to fill them in subsequent contacts with new research participants, this interactive process will involve revisiting the data or returning and forth repeatedly on the data [19]. Sub-themes and main themes will be used to group the transcribed text. The coding and category creation will be carried out by two separate investigations, and disagreements will be settled through discussion until an agreement is reached. The themes from various stakeholders and adolescents will be compared to gain a more thorough understanding of the concerns and needs for sexual health education needed for adolescent pregnancy prevention, as well as their perceptions of the development of strategies to improve sexual health education strategies that can be used for adolescent pregnancy prevention.

## Discussion

This is the first study to develop and validate strategies to enhance sexual health education for prevention of teenage pregnancy from different Stakeholder’s perspectives in Vhembe district, in Limpopo Province. The study will assist in exploring misconceptions and myths sexual health education that may be used for prevention of adolescent pregnancy and may provide a platform to address the concerns and clarify myths as well as address the knowledge gap. It can also provide adolescents and community members with an opportunity to rethink their stance regarding their influence in the prevention of adolescent pregnancy. It will help to assess the gap between their sexual and reproductive health education needs and the services provided to them regarding the prevention of adolescent pregnancy related matters. It will shed more light and better understanding on sexual health education practices that must be used to enhance adolescents as well as their needs and preferences, and that will help in developing the strategies that will be effective in reducing the number of unwanted pregnancies. If the practice of sexual health education strategies is effective it will lead to more young people finishing their studies and affording them better chance in getting jobs. It will also, inform the implementation of these strategies to test their effectiveness in this context another context.

### Supplementary Information

**Additional file 1**: Informed consent. 

**Additional file 2**: Assent form. 

**Additional file 3**: Focus group discussion guide. 

## Data Availability

The materials used in this paper are only related to the study protocol, and no new data are reported. The datasets will be collected and analyzed, and the corresponding author will make them available upon reasonable request. Additional file 3 contain focus group guides developed for this study protocol.

## Declarations

### Study timeline

Data collection will take place between September 2023 to February 2024. The data analysis will take place concurrently with data analysis and this will happen after a moth of each time we have collected data for each phase, and the results will be released shortly after.
