# Correction: Strategies to enhance sexual health education for prevention of teenage pregnancy in Vhembe District, Limpopo Province: different stakeholder’s perspectives, a co-operative inquiry qualitative protocol paper

**DOI:** 10.1186/s12978-023-01689-7

**Published:** 2023-10-06

**Authors:** Nombulelo V. Sepeng, Fhumulani M. Mulaudzi, Pfarelo Mathivha, Maurine R. Musie, Raikane J. Seretlo

**Affiliations:** 1 Department of Nursing Science, Faculty of Health Sciences, University of Pretoria, Tshwane, South Africa; 2 Department of Public Health, Faculty of Health Science, Sefako Makgatho University, Tshwane, South Africa

**Correction: Reproductive Health (2023) 20:120** **https://doi.org/10.1186/s12978-023-01669-x**

After publication of this article [1], the authors reported that the author name ‘Fhumulani M. Mulaudzi’ was incorrectly written as ‘Fhmulani M. Mulaudzi’, and the author name ‘Maurine R. Musie’ was incorrectly written as ‘Maurine F. Musie’.

The original article [1] has been corrected.
