# Supplementary material for: Strategies to enhance sexual health education for prevention of teenage pregnancy in Vhembe District, Limpopo Province: different stakeholder’s perspectives, a co-operative Inquiry qualitative protocol paper
Source: Reprod Health. 2023 Aug 18;20:120. doi: 10.1186/s12978-023-01669-x (PMC10439615; doi:10.1186/s12978-023-01669-x)
Supplement: Supplementary file 1 — Additional file 1: Informed consent. [file 12978_2023_1669_MOESM1_ESM.docx]

**ANNEXURE B-CONSENT FORM**

# ICD 4

# PARTICIPANT’S INFORMATION AND INFORMED CONSENT DOCUMENT FOR A

#

# FOCUS GROUP INTERVIEW RESEARCH STUDY

**Study title: Strategies to enhance sexual health education for prevention of teenage pregnancy in Venda: Different Stakeholder’s perspectives**

**Principal Investigator:** Dr NV Sepeng

**Other Investigators:** DR Musie M.R, Prof. FM Mulaudzi, Ms Mathivha, P , Mr RJ Seretlo

**Institution: University of Pretoria**

**DAYTIME AND AFTER-HOURS TELEPHONE NUMBER(S):**

**Daytime number/s: 071 923 6362**

**After hours number:**

**Date and time of informed consent discussion:**

|  |  |  |  | **:** |
| --- | --- | --- | --- | --- |
| **date** | **Month** | **Year** |  | **Time** |

**Dear Prospective Participant**

**Dear Mr. / Mrs. ...............................**

**Introduction**

You are invited to volunteer for a research study. The project will be conducted in Vhembe District, in Vuwani through collaboration with ISA Mathivha HOPE NGO. This document gives you information to help you decide if you would like to participate. Before you agree to take part in this study you should fully understand what is involved. If you have any questions, which are not fully explained in this document, do not hesitate to ask the investigator. You should not agree to take part unless you are completely happy about what we will be discussing during the focus group discussion.

**The aim of this study**

The aim of this study is to develop stakeholder’s informed sexual health education strategies that can be used for prevention of teenage pregnancy in Venda following a Co-operative Inquiry (CIG). This stakeholder’s informed sexual health education strategies involve information and good that can be used by teenagers to prevent teenage pregnancy in Vhembe district. Those practices entail the practices such as teaching the teenagers and having dialogue regarding the sexual health information for prevention of teenage pregnancy, sexual and reproductive age, the importance of mentorship, importance of delaying and abstain from sexual intercourse from young age, and the implications of teenage pregnancy. Also, practices such as the use of condoms and contraceptives that can be used by teenagers to prevent teenager’s pregnancy and support measures available for pregnant teenagers.

**Procedures to be followed**

This study will be conducted following phases of CIG that is reflection, planning, action and observation. Focus group discussion will be used as a method of data collection. If you are to participate in the study the discussion will be arranged at a time that is convenient to you, with other teenagers. You will be 8 to 10 in each group. The focus group discussions will take place face to face in ISA foundation Non-Governmental agency in Vuwani. For those, who are under the age of 16, we will need their parents to give us the permission to take part in the study and the informed shared with us will not be stared to your parents in any case. Focus group discussions may last to one hour. The study will take place into three phases. In the first phase, the researchers will explore and describe to explore and describe concerns and educational needs of adolescents regarding the sexual health education for prevention of teenage pregnancy in Venda. Also, the researchers will explore and describe the perceptions of adolescents regarding the development of strategies to enhance sexual health education for prevention teenage pregnancy in Venda. After that the researchers will analyse data and setup the meeting with you to present the findings that will be used to start with phase 2. In phase 2, the researchers will ask questions regarding what information may be used to develop strategies to enhance sexual health education for prevention of teenage pregnancy in Venda. After that the researchers will analyse data and setup the meeting with you to present the findings that will be used to start with phase 3. Lastly in phase 3, the researchers ask questions that will be used to evaluate the strategies used to enhance sexual health education for prevention of teenage pregnancy and this will be the final phase of the study.

**Risks involved**

We do not think that taking part in the study will cause any physical or emotional discomfort or risk. You do not have to share any knowledge you are not comfortable with. If questions feel too personal or make you uncomfortable, you do not have to answer them. If you need psychological support or counselling during or after the interviews. I will be able to refer you to a social worker working for HOPE NGO for professional counselling, the name of the counsellor is Mrs Pfarelo Mathihva, is working in collaboration with the researcher and she agreed to offer counselling if the need be, her cell numbers are 076 414 8541.

**Benefits involved**

The envisaged benefit is that the study will identify gaps regarding the intervention that will be used for prevention of teenage pregnancy.

You will not be paid to take part in the study. There are no costs involved for you to be part of the study. The decision to take part in the study is yours and yours alone. You do not have to take part if you do not want to. You can also stop at any time during the interview without giving a reason. If you refuse to take part in the study, this will not affect you in any way.

This study was submitted to the Research Ethics Committee of the Faculty of Health Sciences at the University of Pretoria, Medical Campus, Tswelopele Building, Level 4-59, telephone numbers 012 356 3084 / 012 356 3085 and written approval has been given by that committee. The study will follow the Declaration of Helsinki (last update: October 2013), which guides doctors on how to do research in people. The researcher can give you a copy of the Declaration if you wish to read it.

If you have any questions concerning this study, you should contact the researcher (Dr NV Sepeng 071 923 6362).

**10. CONFIDENTIALITY**

We will not record your name anywhere and no one will be able to connect you to the answers you give. Your answers will be linked to a fictitious code number or a pseudonym (another name) and we will refer to you in this way in the data, any publication, report or other research output. All records from this study will be regarded as confidential. Results will be published in medical journals or presented at conferences in such a way that it will not possible for people to know that you were part of the study.

All hard copy information will be kept in a locked facility in the Department of Nursing Sciences at the University of Pretoria, for a minimum of 15 years and only the research team will have access to this information.

- I confirm that the person requesting my consent to take part in this study has told me about the nature and process, any risks or discomforts, and the benefits of the study.
- I have also received, read and understood the above written information about the study.
- I have had adequate time to ask questions and I have no objections to participate in this study.
- I am aware that the information obtained in the study, including personal details, will be anonymously processed and presented in the reporting of results.
- I understand that I will not be penalized in any way should I wish to discontinue with the study and my withdrawal will not affect my treatment and care.
- I am participating willingly.
- I have received a signed copy of this informed consent agreement.

__________________________________ ________________________

Participant’s name (Please print) Date

__________________________________ ________________________

Participant’s signature Date

__________________________________ ________________________

Researcher’s name (Please print) Date

__________________________________ ________________________

Researcher’s signature Date

I understand that the focus group discussion will be audiotaped. I give consent that it may be audio recorded.

YES

NO

__________________________________ ________________________

Participant’s name (Please print) Date

__________________________________ ________________________

Participant’s signature or thumbprint Date

__________________________________ ________________________

Investigator's name (Please print) Date

__________________________________ ________________________

Investigator's signature Date

__________________________________ ________________________

Name of the person who witnessed

the informed consent (Please print) Date

__________________________________ ________________________

Signature of the witness Date
