# Supplementary material for: Strategies to enhance sexual health education for prevention of teenage pregnancy in Vhembe District, Limpopo Province: different stakeholder’s perspectives, a co-operative Inquiry qualitative protocol paper
Source: Reprod Health. 2023 Aug 18;20:120. doi: 10.1186/s12978-023-01669-x (PMC10439615; doi:10.1186/s12978-023-01669-x)
Supplement: Supplementary file 2 — Additional file 2: Assent form. [file 12978_2023_1669_MOESM2_ESM.docx]

# INFORMATION AND ASSENT DOCUMENT FOR 15 -18 YEARS

**Study title:** **Strategies to enhance sexual health education for prevention of teenage pregnancy**

**in Venda: Different Stakeholder’s perspectives**

**Principal Investigator: Dr NV Sepeng**

**Other investigators:** Prof FM Mulaudzi, Dr MR Musie, Ms P Mathivha and Mr RJ Seretlo

**Institution: University of Pretoria**

**Daytime telephone number/s: 071 923 6362**

**Date and time of informed consent discussion:**

|  |  |  |  | **:** |
| --- | --- | --- | --- | --- |
| **Date** | **Month** | **Year** |  | **Time** |

1. **INTRODUCTION**

My name Dr NV Sepeng and we will conducting a study/research among children, especially adolescents. We want to know how their views regarding their concerns and sexual health education needs for prevention of teenage pregnancy and their perceptions regarding the development of strategies to enhance sexual health education for prevention teenage pregnancy in Venda.

I am going to explain this research to you and invite you to be part of this research study. You can choose whether or not you want to participate in this study. We have discussed this research study with your parent, the teachers and the area chief and they know that we are also asking for your permission. If you are going to be part in this research, you will sign this form. But if you do not wish to participate, you do not have to.

You may discuss anything on this form with your parents, teachers or friends. You can decide whether to participate or not after you have talked it over. You do not have to decide immediately. There may be some words you don't understand or things that you want me to explain to you. Please ask me to stop at any time and I will explain.

1. **WHAT IS RESEARCH?**

Research is what we do to find new knowledge about subjects (and people). We use research studies to help us find more information about disease or illness. Research also helps us to find better ways of treating children who are sick.

1. **WHAT IS THIS RESEARCH PROJECT ALL ABOUT AND WHAT IS EXPECTED OF ME?**

Children, especially adolescents often fall pregnant unplanned and are faced with the consequences. We want to develop of strategies to enhance sexual health education for prevention teenage pregnancy in Venda. We need information about your views about the existing interventions in place for prevention of adolescent pregnancy, we also want to know your perceptions regarding the development of strategies to enhance sexual health education for prevention teenage pregnancy in Venda.

1. **WHY HAVE I BEEN INVITED TO TAKE PART IN THIS RESEARCH PROJECT?**

You are a child aged between 16-19 years. This makes you an adolescent exposed to teenage pregnancy.

1. **WHO IS DOING THE RESEARCH?**

The researchers are health care professionals.

1. **WHAT WILL HAPPEN TO ME IN THIS STUDY?**

We are going to ask questions in a group and individually. You are going to participate in focus group discussions regarding your concerns and educational needs of adolescents regarding the sexual health education for prevention of teenage pregnancy and your perceptions regarding the development of strategies to enhance sexual health education for prevention teenage pregnancy in Venda. And in the development of these interventions.

1. **CAN ANYTHING BAD HAPPEN TO ME?**

Nothing bad can happen to you because of this research study.

1. **CAN ANYTHING GOOD HAPPEN TO ME?**

You will improve your interpersonal skills, problem solving skills and working effectively in a team.

1. **ETHICS APPROVAL**

This Protocol was submitted to the Faculty of Health Sciences Research Ethics Committee, University of Pretoria, Medical Campus, Tswelopele Building, Level 4-59, Telephone numbers 012 356 3084 / 012 356 3085 and written approval has been granted by that committee.

1. **WILL ANYONE KNOW I AM IN THE STUDY?**

Only the study personnel will know that you are participating in the study.

1. **Who can I talk to about the study?**

Dr Nombulelo Veronica Sepeng, 071 923 6362

1. **WHAT IF I DO NOT WANT TO DO THIS?**

You do not have to participate in the study, even if your parent have signed consent that you can participate.

You can also withdraw from the study at any time without getting in trouble.

You must just remember that participating in this study will help prevent adolescent pregnancy and its consequences.

1. **CONSENT TO PARTICIPATE IN THIS STUDY**

Do you understand this research study and are you willing to participate in it?

| YES |  | NO |
| --- | --- | --- |

Do you understand what is expected of you as participant?

| YES |  | NO |
| --- | --- | --- |

Has the researcher answered all your questions?

| YES |  | NO |
| --- | --- | --- |

Do you understand that you can pull out of the study at any time without any consequences?

| YES |  | NO |
| --- | --- | --- |

You don’t have to give us your answer now, take your time and read the rest of this form before you decide.

If you sign at the bottom it will mean that you have read this paper, and that you would like to be in this study.

|  | **Your Name** | **Person Obtaining**  **Consent** | **Principle** |
| --- | --- | --- | --- |
| **Name**  ^Please Print^ |  |  |  |
| **Signature** |  |  |  |
| **Date** |  |  |  |
