# Supplementary material for: Strategies to enhance sexual health education for prevention of teenage pregnancy in Vhembe District, Limpopo Province: different stakeholder’s perspectives, a co-operative Inquiry qualitative protocol paper
Source: Reprod Health. 2023 Aug 18;20:120. doi: 10.1186/s12978-023-01669-x (PMC10439615; doi:10.1186/s12978-023-01669-x)
Supplement: Supplementary file 3 — Additional file 3: Focus group discussion guide. [file 12978_2023_1669_MOESM3_ESM.docx]

**ANNEXURE C: FOCUS GROUP DISCUSSION GUIDE**

The researcher is going to have focus group session with the teenagers and stakeholders in three phases. The researcher will use open-ended questions to explore and describe to explore and describe teenagers concerns and sexual health education needs for prevention of teenage pregnancy in Venda. To explore and describe the perceptions of teenagers regarding the development of strategies to enhance sexual health education for prevention teenage pregnancy in Venda. To develop strategies to enhance sexual health education for prevention of teenage pregnancy in Venda. Lastly to evaluate the strategies used to enhance sexual health education for prevention of teenage pregnancy

**Questions**

**Section A: Demographic data**

- How old are you?
- What gender are you?
- What is your religion?
- Level of education/ what grade are you doing?
- What is your profession

**Section B: Questions for focus group**

***Phase one: Reflection questions and planning questions***

1. What are the current strategies in place used for teenage pregnancy in Venda?
2. What are your concerns regarding teenage pregnancy interventions in place?
3. What are your educational needs regarding teenage pregnancy interventions in place?
4. What are your perceptions regarding the development of strategies to enhance sexual health education for prevention teenage pregnancy?

***Phase two: Action questions***

1. What is the information that must be included for the development of strategies to enhance the prevention of teenage pregnancy?
2. Which stakeholders do you think must provide sexual health education to enhance the prevention of teenage pregnancy?
3. What is the role of stakeholders that must provide sexual health education to enhance the prevention of teenage pregnancy?
4. What do you think will make these strategies different from the current interventions?

***Phase three: Observation evaluation questions***

1. Do you think all the information that you have suggested for the development of strategies captured as it is?
2. What do you think could be a barrier for the implementation of these strategies to enhance sexual health education for prevention teenage pregnancy?
3. What do you think could be facilitating factors for the implementation of these strategies to enhance sexual health education for prevention teenage pregnancy?
4. Are there any changes that you want to add or remove?

Probing questions will be used as the need arise for further clarity, paraphrasing and summarizing will also be used to reflect on the participants. The interviews will be audio/video- recorded with the participant’s permission. Field notes will be taken on the non- verbal responses of the participants by a co-researcher.
